# Comparison of SARS-CoV-2 variants of concern in primary human nasal cultures demonstrates Delta as most cytopathic and Omicron as fastest replicating

**DOI:** 10.1128/mbio.03129-23

**Published:** 2024-03-13

**Authors:** Nikhila S. Tanneti, Anant K. Patel, Li Hui Tan, Andrew D. Marques, Ranawaka A. P. M. Perera, Scott Sherrill-Mix, Brendan J. Kelly, David M. Renner, Ronald G. Collman, Kyle Rodino, Carole Lee, Frederic D. Bushman, Noam A. Cohen, Susan R. Weiss

**Affiliations:** 1 Department of Microbiology, University of Pennsylvania Perelman School of Medicine, Philadelphia, Pennsylvania, USA; 2 Department of Otorhinolaryngology- Head and Neck Surgery, Perelman School of Medicine, University of Pennsylvania, Philadelphia, Pennsylvania, USA; 3 Department of Medicine, Perelman School of Medicine, University of Pennsylvania, Philadelphia, Pennsylvania, USA; 4 Corporal Michael J. Crescenz VA Medical Center, Surgical Services, Philadelphia, Pennsylvania, USA; 5 Monell Chemical Senses Center, Philadelphia, Pennsylvania, USA; Washington University in St. Louis, St. Louis, Missouri, USA; Johns Hopkins University Bloomberg School of Public Health, Baltimore, Maryland, USA

**Keywords:** SARS-CoV-2, COVID-19, variants of concern, primary nasal cultures, spike cleavage, dsRNA

## Abstract

The SARS-CoV-2 pandemic was marked with emerging viral variants, some of which were designated as variants of concern (VOCs) due to selection and rapid circulation in the human population. Here, we elucidate functional features of each VOC linked to variations in replication rate. Patient-derived primary nasal cultures grown at air-liquid interface were used to model upper respiratory infection and compared to cell lines derived from human lung epithelia. All VOCs replicated to higher titers than the ancestral virus, suggesting a selection for replication efficiency. In primary nasal cultures, Omicron replicated to the highest titers at early time points, followed by Delta, paralleling comparative studies of population sampling. All SARS-CoV-2 viruses entered the cell primarily via a transmembrane serine protease 2 (TMPRSS2)-dependent pathway, and Omicron was more likely to use an endosomal route of entry. All VOCs activated and overcame dsRNA-induced cellular responses, including interferon (IFN) signaling, oligoadenylate ribonuclease L degradation, and protein kinase R activation. Among the VOCs, Omicron infection induced expression of the most IFN and IFN-stimulated genes. Infections in nasal cultures resulted in cellular damage, including a compromise of cell barrier integrity and loss of nasal cilia and ciliary beating function, especially during Delta infection. Overall, Omicron was optimized for replication in the upper respiratory tract and least favorable in the lower respiratory cell line, and Delta was the most cytopathic for both upper and lower respiratory cells. Our findings highlight the functional differences among VOCs at the cellular level and imply distinct mechanisms of pathogenesis in infected individuals.

**IMPORTANCE:**

Comparative analysis of infections by SARS-CoV-2 ancestral virus and variants of concern, including Alpha, Beta, Delta, and Omicron, indicated that variants were selected for efficiency in replication. In infections of patient-derived primary nasal cultures grown at air-liquid interface to model upper respiratory infection, Omicron reached the highest titers at early time points, a finding that was confirmed by parallel population sampling studies. While all infections overcame dsRNA-mediated host responses, infections with Omicron induced the strongest interferon and interferon-stimulated gene response. In both primary nasal cultures and lower respiratory cell line, infections by Delta were most damaging to the cells as indicated by syncytia formation, loss of cell barrier integrity, and nasal ciliary function.

## INTRODUCTION

The SARS-CoV-2 pandemic has been marked by evolution of the ancestral virus strains, Wuhan in the east and Washington in the west, into new variants. The World Health Organization has identified some of the variants that pose an increased risk for global public health as variants of concern (VOCs). Characteristics for VOCs include an increase in virus transmissibility and virulence, and/or a decrease in response to current vaccines, public health measures, and therapeutics (1).

Four major VOCs have emerged over the course of the pandemic. The Alpha VOC, B.1.1.7 Pango lineage virus, was first documented in the United Kingdom in September 2020. The Beta VOC, B.1.351 Pango lineage virus, was first documented in South Africa in May 2020. The Delta VOC, B.1.617.2 Pango lineage virus, was first identified in India in October 2020. The B.1.1.529 Pango lineage Omicron variant was first documented in South Africa in November 2021 (2); however, more recent reports suggest an earlier emergence. Since then, several sub-variants have emerged from the Omicron lineage, including the BA.5 and XBB.1.5, which have quickly established global dominance and, finally, BQ.1 in some regions (3).

Clinical retrospective studies report differences in patient outcomes among VOCs. Compared to the ancestral strain, patients infected with Delta and Alpha variants experienced heightened disease severity, such as an increase in oxygen requirement, longer hospitalization, and morbidity (4). Patients infected with the Omicron variant were less likely to develop severe COVID-19, require hospitalization, and had lower rates of in-hospital mortality compared to Delta-infected patients, but the assessment of these traits was confounded by the concomitant development of immunity from prior exposure and vaccination in these populations (5, 6).

Variant-specific mutations to the ancestral SARS-CoV-2 genome are of interest for their potential roles in facilitating virus pathogenesis and spread. Many of the amino acid substitutions are found in the spike-encoding region of the SARS-CoV-2 genome. The spike (S) protein, composed of S1 and S2 subunits, binds to the host angiotensin-converting enzyme 2 (ACE2) receptor to facilitate virus-host membrane fusion, virus entry, and modulates host immune responses (7–9). Several studies attribute the enhanced immune evasion by the variants to substitutions in the spike protein. Amino acid substitutions that render the furin protease recognition site at the spike S1/S2 subunit junction more basic have been associated with enhanced viral fitness for the Delta VOC and, to a lesser extent, for the Alpha and Omicron variants (10). Deletions and substitutions within and adjacent to the furin protease recognition site have been associated with virus attenuation (11, 12). The role of mutations outside of the spike gene, although not as well characterized, likely also contributes to differences in pathogenesis. For example, mutations in the nucleocapsid gene have been associated with increased virus replication and pathogenesis (13).

To assess genome-wide differences between SARS-CoV-2 VOCs in a controlled system, we compared molecular replication mechanism among full length, replication competent Alpha, Beta, Delta, and Omicron VOCs to the ancestral Washington (WA1). All viruses were compared for replication kinetics and cellular responses to infections in patient-derived primary nasal cultures, with the goal of modeling the first step of infection. In addition, all experiments were repeated in cell lines derived from human lung tissues, including Calu-3 and A549, to facilitate more mechanistic studies that are not limited by primary cells. The cell lines also provide the context of a lower respiratory model for infections. Our experiments clarify differences among variants in virus entry, virus replication, cell-to-cell spread of the virus, and activation of host innate immune responses and antiviral pathways.

This study adds to our understanding of SARS-CoV-2 variants in several ways. We compared the ancestral WA1 to Alpha, Beta, Delta, and Omicron variants, while many focus on a subset of this group. All experiments involve infections with authentic viruses in a BSL3 facility rather than the use of pseudoviruses or protein expression systems. In addition to quantifying RNA copy numbers, which can be misleading with RNA viruses, our experiments also quantify infectious viruses. While several useful animal models have been developed to study SARS-CoV-2 (14, 15), we find that our patient-derived nasal epithelia model more faithfully reflects replication kinetics in the human nose, the first site of infection.

## RESULTS

### VOCs exhibit increased replication in upper respiratory cells

**Fig 1:**
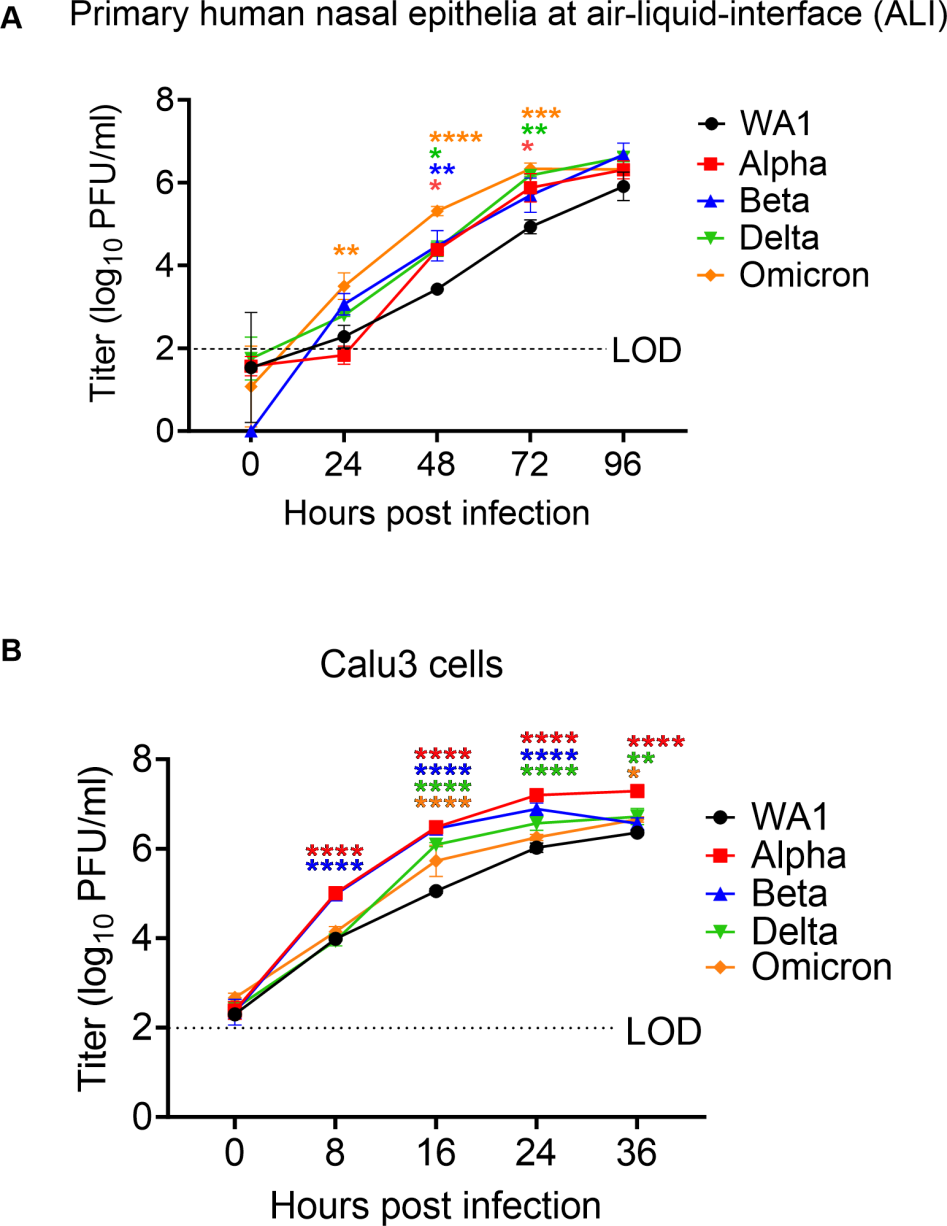
Replication of SARS-CoV-2 WA1 and VOCs. (A and B) Human primary nasal epithelia ALI cultures (**A**) or Calu-3 cells (**B**) were infected with SARS-CoV-2 WA1, Alpha, Beta, Delta, and Omicron at MOI 0.1 PFU/cell, and apically shed virus was titered by plaque assay at indicated hours post infection. (**A**) Titers from nasal cultures are an average of three independent experiments, each experiment was performed with three individual donor cells totaling to nine individual donors per virus. (**B**) Titers from Calu3 are an average of two independent experiments each in triplicates. (**A and B**) Graphed values represent mean with standard deviation, and statistics for both A and B were performed with ordinary two-way ANOVA comparing VOCs versus WA1 within a time point, multiple comparisons adjusted *P*-values: *P < 0.01, **P < 0.001, ***P < 0.0001, ***P < 0.00001. LOD, limit of detection, by plaque assay.

SARS-CoV-2 infections are initiated in the upper respiratory tract, so we sought to model this step by comparing infections in primary cells collected from nasal cavities of patients undergoing rhinologic evaluation. These cells were cultured on transwells at an air-liquid interface (ALI) to recapitulate the natural state of the nasal epithelium, as we have reported previously (16). In virus growth curve assays, following infection of primary nasal cultures at a low multiplicity of infection (MOI = 0.1), all SARS-CoV-2 viruses replicated to high titers (>1 × 10^5^ PFUs/mL), and all VOCs replicated to higher titers than WA1 (Fig. 1A). Omicron reached the highest titers at early time points of infection, starting at 24 hours post infection (hpi), and maintained the highest titer until 72 hpi, after which titers start to drop. WA1 generally produced the lowest titers compared to all variants. The observation that late variants reach higher titers than early variants in primary nasal cultures suggests that emerging variants have been selected for replication in human nasal epithelium. The growth curve experiment was ended at 96 hpi as the infected nasal cultures began to die, and titers were no longer significantly different at this point.

Coronavirus infections naturally progress from the upper to the lower respiratory tract. To investigate the relative efficiencies of replication of the variants in cells of the lower respiratory tract, we used the Calu3 cell line derived from human lung epithelia. Similar to observations in the primary nasal cultures, all VOCs replicated to higher titers than the ancestral WA1 in Calu3, suggesting that all VOCs were selected for more efficient replication than the ancestral SARS-CoV-2 (Fig. 1B). However, the Alpha and Beta VOCs reached significantly higher titers than Omicron (at 8 hpi) in Calu3 infections, while Omicron reached significantly higher titers than Alpha/Beta/Delta in nasal cultures. Also, all viruses reached peak titers at an earlier time (24–36 hpi) in Calu3 cells than in nasal cultures. As the Calu3 cells begin to show signs of cytopathic effect and cell death around 36 hpi, the experiment could not be extended to further duration. Together, these results suggest that while all VOCs replicate more efficiently than the ancestral virus, Omicron is especially selected for heightened replication in nasal cultures.

To ensure the integrity of the experiments, genomic RNA from each virus stock was sequenced and aligned against the Washington A reference genome before further analysis (Fig. S1A). The alignment confirmed that all viruses used in this study maintain the defining mutations of each lineage. Additionally, it confirmed that no new substitutions have become fixed at the known hotspots, including the furin protease recognition site (11).

The COVID-19 pandemic exhibited waves of illnesses in the colder months, suggesting a seasonal pattern similar to other respiratory viruses (17). Replication fitness at different temperatures may impact the potential for VOCs to cause seasonal outbreaks. To investigate whether the VOCs have adapted to colder temperatures, similar to the seasonal common cold coronaviruses, infections with WA1, Delta, and Omicron viruses were performed in nasal cultures at 33°C and 37°C (Fig. S3A). We did not observe any significant differences in titer when comparing infections at both temperatures up to 96 hpi, suggesting that SARS-CoV-2 replication is not temperature sensitive early in infection. However, preference for WA1 at 33°C at later times post infection has been reported (16).

### The influence of furin protease cleavage of spike on viral entry and cell-to-cell spread

**Fig 2:**
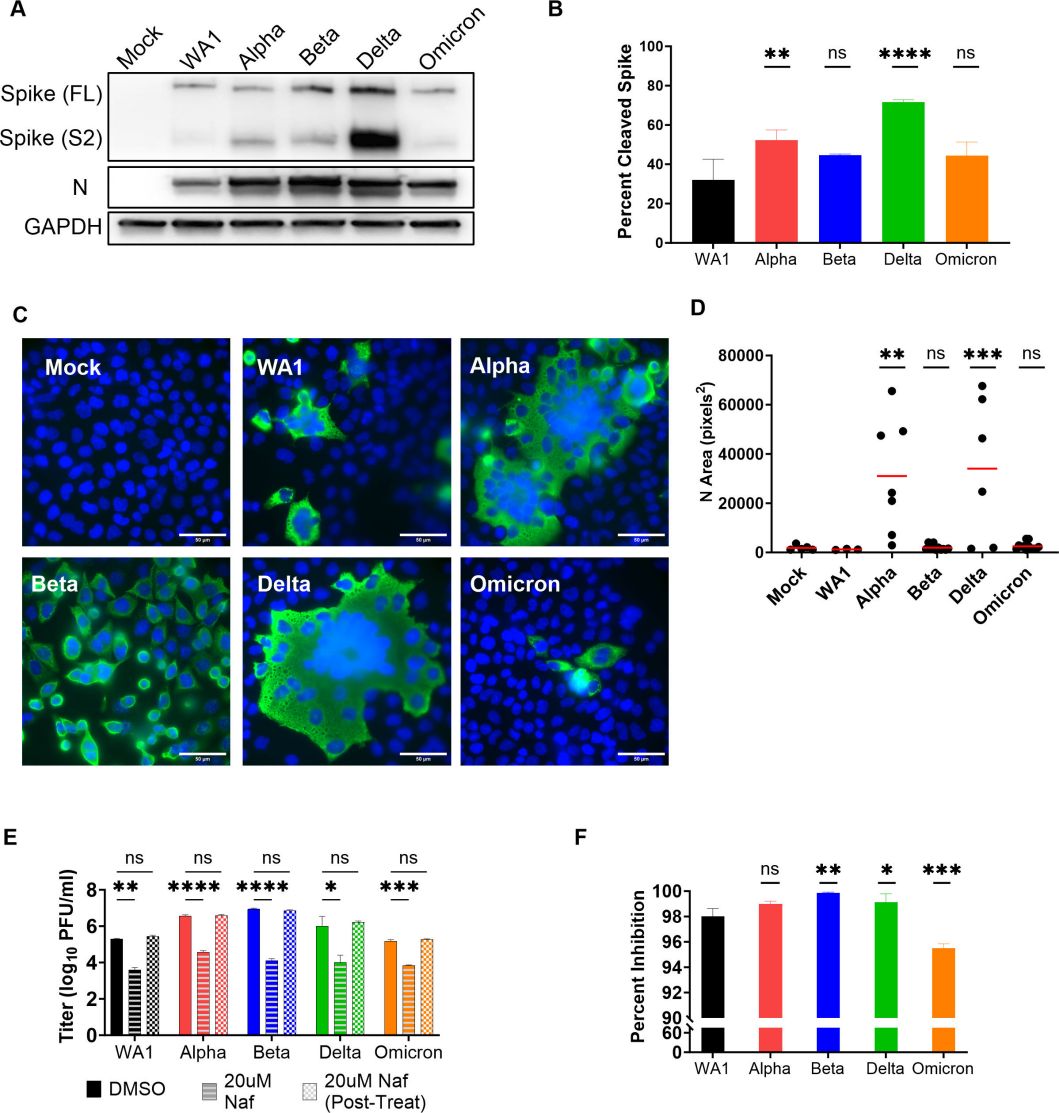
Virus entry and spread. (**A**) Protein lysates from nasal cultures infected with SARS-CoV-2 WA1 and VOCs were analyzed by polyacrylamide gel electrophoresis followed by immunoblotting with antibodies against SARS-CoV-2 proteins, nucleocapsid (N) and spike S2, which recognize the full length (FL) and cleaved (S2) forms. Cellular protein GAPDH was used for loading control of the gels. Western blot depicted is representative of three independent donor infections, and these protein lysates are also used in Fig. 6A. (**B**) Percent cleaved spike was calculated using the following formula: S2/(FL + S2). Graphed values are an average from three independent western blots, and statistics were performed with ordinary one-way ANOVA comparing VOC against WA1, adjusted *P*-values: **P < 0.001, ****P < 0.00001, ns, not significant. (**C**) A549^ACE2^ cells infected with SARS-CoV-2 WA1 and VOCs (MOI = 0.01 for 24hpi), fixed, and stained with fluorescently labeled antibodies DAPI (blue) and SARS-CoV-2 nucleocapsid (green). Images are representative of three independent infections. (**D**) Independent clusters expressing N were quantified for area (pixel^2^) from three independent experiments. Graph represents individual values with mean in red, and statistics were performed with two-way ANOVA with multiple comparisons of VOCs versus WA1, adjusted P-values: **P < 0.001, ***P < 0.0001, ns, not significant. (**E**) Calu3 cells were mock treated (DMSO), pre-infection treated with 20 uM Nafamostat (Naf) for 2 hours, or pre- and post-infection treated with 20 uM Naf for 2 hours. Infections with SARS-CoV-2 WA1 and VOCs were performed at MOI = 0.1, and shed virus was collected at 16 hpi for titer by plaque assay. Graphed values are an average of two independent infections each performed with biological triplicates. Statistics were performed with two-way ANOVA comparing 0 uM to Nafamostat treatments, *P*-values: *P < 0.01, **P < 0.001, ***P < 0.0001, ****P < 0.00001, ns, not significant. (**F**) Percent inhibition in virus titer after Naf treatments. Graphed values represent mean with standard deviation, and statistics were performed with ordinary one-way ANOVA comparing the VOC against WA1, adjusted *P*-values: *P < 0.01, **P < 0.001, ***P < 0.0001, ns, not significant.

To understand the mechanisms of increased replication of the VOCs, we investigated various factors that contribute to replication such as virus spike cleavage (Fig. 2A and B), cell-to-cell spread (Fig. 2C and D), and route of entry (Fig. 2E and F). Cleavage of the spike protein at the furin recognition site facilitates both virus entry and cell-to-cell virus spread, a mechanism that could contribute to increased replication of the variants. The basic amino acids at the furin recognition site of the spike protein, PRRAR in WA1, recruit proteases necessary for the cleavage at the S1/S2 junction (18). This region is sensitive to substitutions which influence infection and replication (11, 19–21). We hypothesized that the amino acid substitutions in VOCs that render the furin cleavage site more basic would be more efficiently cleaved, leading to increased cell-to-cell spread. Specifically, we hypothesized that Delta, which encodes the most basic amino acids in the cleavage-site (RRRAR), will generate the most cleaved spike, followed by Alpha and Omicron (both HRRAR) generating equal levels of cleaved spike. Beta and WA1, which encode an S1/S2 site with the least basic charge (PRRAR), were expected to generate the least cleaved spike.

To measure the ratios of cleaved versus full-length spike protein levels among VOCs, a western blot was performed using protein lysates from infected primary nasal cultures (Fig. 2A). As predicted, quantification of the cleaved spike from three separate donors showed that Delta infections generated the highest proportion of cleaved spike (71%), followed by Alpha infection (52%), Omicron and Beta infections generated similar levels of cleaved spike (~44% each), and the lowest by WA1 infection (32%) (Fig. 2B). The elevated levels of cleaved spike in infections by Alpha and Delta compared to WA1 are consistent with the idea that these VOCs have evolved to optimize spread in human nasal cells. However, infection with Omicron produced the highest titers (Fig. 1A) despite inefficient spike cleavage (Fig. 2A and B), indicating the importance of factors affecting other replication steps.

A similar experiment in the VeroE6^TMPRSS2^ cell line yielded different relative ratios of cleaved spike among VOCs (Fig. S2A). In this western blot, protein lysates from infected VeroE6^TMPRSS2^ cells were analyzed, using equal levels of full-length spike to visualize differences in the ratios of cleaved spike among the VOCs. While the highest ratio of cleaved spike was produced with Delta infection (67% cleaved) as predicted and observed by Khatri et al. (22), contrary to our hypothesis, infection with the Beta generated the second most cleaved spike. Infections with Alpha and Omicron VOC generated different levels of cleaved spike despite sharing the same furin cleavage site sequence, similar to observations of spike proteins from primary nasal cultures. These results add to our understanding that while the strong basic charge at the furin cleavage facilitates spike cleavage, there are other factors beyond the PRRAR sequence that facilitate cleavage. In addition, cell type-specific biology, e.g., variation in the abundances of various proteases, may influence the cleavage of spike. As a validation of our spike cleavage assay, we included infections with a positive control virus (icWT), expressing a spike containing the ancestral PRRAR sequence and a negative control virus (ic∆PRRA), expressing a spike with a deletion of the PRRA sequence, both generated with the infectious clone reverse genetics system (Fig. S2A) (11). As expected, infections with both WA1 and icWT generated comparable ratios of S2 spike as both viruses encode the same PRRAR furin recognition site. Infection with the ic∆PRRA expressing a spike lacking the PRRA sequence did not generate any cleaved spike (Fig. S2A).

To visualize increased cell-to-cell spread by the VOCs, we used an immunofluorescence staining assay. Due to the heterogenous nature of primary nasal cultures and weakly adhering nature of Calu3 cells, which do not make them ideal for visualizing syncytia, we used a monolayer of A549^ACE2^ cell line for this assay. A monolayer of A549^ACE2^ cells was infected at a low MOI and stained with nucleocapsid and DAPI to visualize syncytia. Compared to WA1, Alpha and Delta infections generated strikingly larger syncytia with the classical clustering of nuclei in the middle (Fig. 2C); syncytia size was quantified by measuring the area of nucleocapsid-positive (green) clusters (Fig. 2D). These data suggest that the Alpha and Delta VOCs are efficient at cell-to-cell spread, likely due to efficient spike cleavage and high fusogenic activity. However, cell-to-cell spread of virus by syncytia formation does not correlate with rate of replication, suggesting the role of different pathways driving these mechanisms.

### SARS-CoV-2 viruses enter primarily by the transmembrane protease serine 2-mediated plasma membrane fusion pathways

During virus entry, the SARS-CoV-2 spike protein is cleaved by TMPRSS2, a serine protease which activates the spike to initiate fusion at the plasma membrane (23–25). It is thought that viruses that express spike with an additional basic amino acid(s) in the furin cleavage recognition site than WA1 (Alpha, Delta, Omicron) would likely use the TMPRSS2-dependent plasma membrane route of infection. However, there are reports of Omicron entering cells in a TMPRSS2-independent endosomal route in some cell types (26–30). To investigate entry via the plasma membrane or endosomal routes, we used the protease inhibitor Nafamostat that blocks TMPRSS2 and inhibits entry by this pathway, which is active in Calu3 cells. Upon treatment of Calu3 cells with Nafamostat, we observed a large reduction in virus titer for all viruses (Fig. 2E). Compared to untreated controls (DMSO), virus titer was inhibited by 95%–99% for all viruses (Fig. 2F), suggesting that all VOCs primarily use the plasma membrane as the major route of entry. Omicron titer was affected the least, suggesting that Omicron is less dependent on the TMPRSS2-mediated entry pathway compared to the other SARS-CoV-2 viruses and, therefore, may use the alternative endosomal route more than other VOCs. Similar results have been reported by others (24, 31–33). However, it is important to note that while Omicron is the least dependent VOC on the plasma membrane fusion pathway, it still enters primarily by this pathway in Calu3 cells.

### SARS-CoV-2 WA1 and VOC plaque morphology

**Fig 3:**
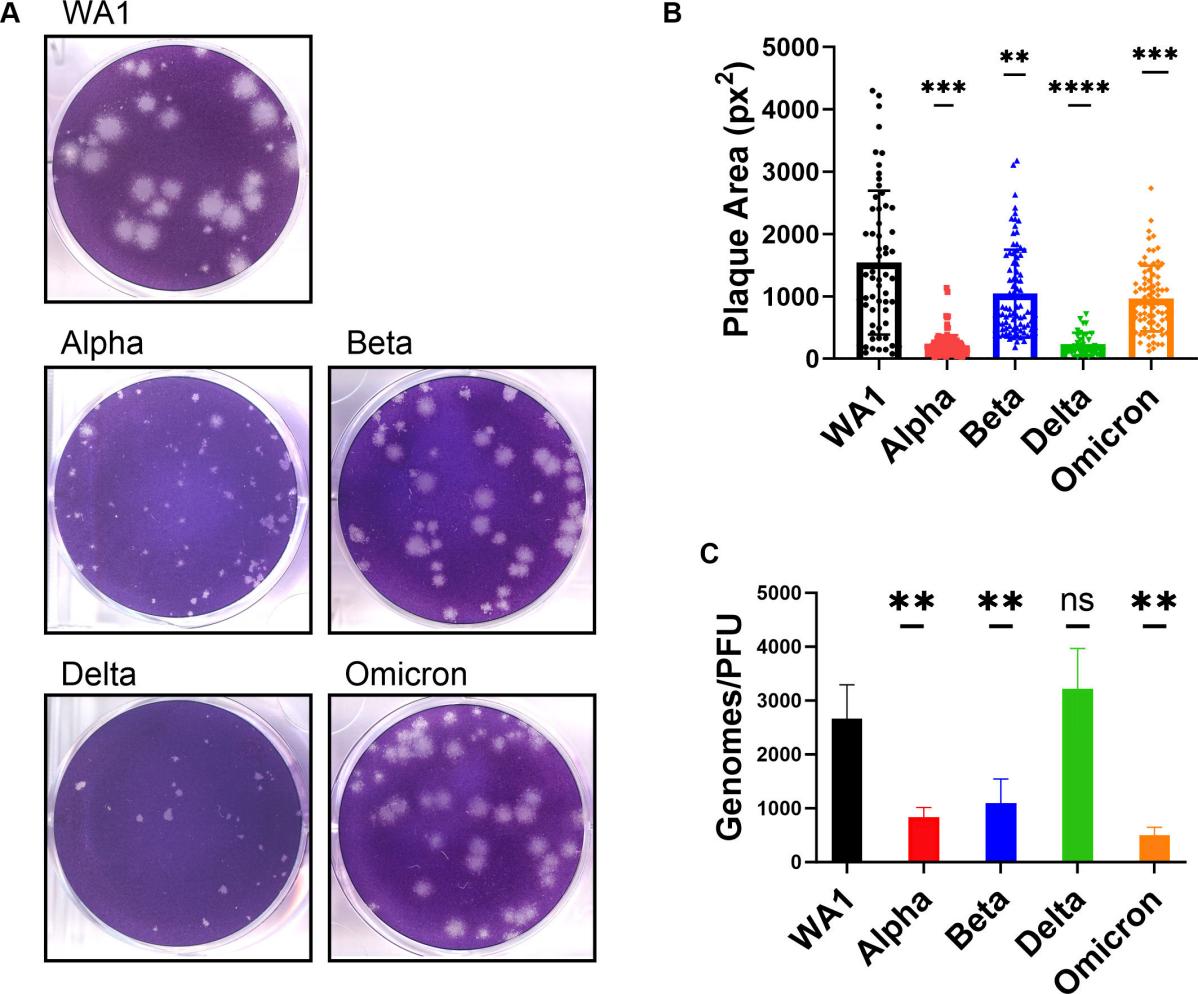
Comparison of plaque size and genome/PFU ratio among WA1 and VOCs.(**A**) Plaque assay of SARS-CoV-2 viruses was performed on VeroE6 cells. (**B**) Three independent plaque assays per virus were quantified for plaque area (pixel^2^), and graph represents the mean of individual plaque areas with standard deviation. Statistics were performed with ordinary two-way ANOVA comparing VOCs versus WA1, adjusted *P*-values: **P < 0.001, ***P < 0.0001, ****P < 0.00001. (**C**) The virus in supernatant collected from Calu3 cells infected at MOI = 0.1 at 24 hpi was assessed for genomes using RT-qPCR with primers specific for SARS-CoV-2 RdRp and compared to PFUs quantified by plaque assay. Values are an average of three independent experiments, each performed in triplicate. Graph represents mean with standard deviation, and statistics were performed with ordinary one-way ANOVA comparing VOCs versus WA1, adjusted *P*-values: **P < 0.001, ns, not significant.

Quantification of infectious virus titer was performed in VeroE6 cells by a plaque assay, during which we observed a variation in plaque morphology and size (Fig. 3A and B). WA1 generated the largest plaques (average 1,542 px^2^) but also displayed the most range in plaque area, as reported by others (34). While all VOCs generated smaller plaques than WA1, the plaques generated with Alpha and Delta VOCs were strikingly smaller (average of 211 and 231 px^2^, respectively). However, it is worth noting that differences in plaque area are abrogated on VeroE6^Tmprss2^-overexpressing cell lines, as reported by others, suggesting a role of cellular protease levels on plaque morphology (35, 36). It is intriguing that, in comparison to WA1, Alpha and Delta generate smaller plaques, even though they produce higher titers of virus in growth curves (Fig. 1A and B) and form larger syncytia (Fig. 2C and D). This demonstrates that for SARS-CoV-2, plaque size does not necessarily correlate with replication and cell-to-cell spread, an observation made previously about murine coronavirus strains (37).

The differences in infectious virus production by titer assays, despite comparable levels of intracellular genome replication by reverse transcriptase-quantitative polymerase chain reaction (RT-qPCR) (Fig. S2B), prompted us to quantify the particle-to-PFU (plaque forming unit) ratio among VOCs. Compared to the WA1 virus, all VOCs, except Delta, secreted significantly fewer genomes per infectious virus (Fig. 3C), suggesting that Alpha, Beta, and Omicron VOCs are more efficient at producing infectious virus. The Delta VOC genome-to-PFU ratio was not significantly different from WA1.

### Omicron infection activates highest levels of interferon and interferon-stimulated genes

dsRNAs generated during the replication of RNA viruses, including coronaviruses, are detected by host cells and elicit antiviral responses. There are three major cytosolic sensors of dsRNAs that induce innate immune responses (38). Detection of coronavirus dsRNA by MDA5 leads to induction of type I and type III interferons (IFN) and activation of interferon-stimulated genes (ISGs), many of which encode proteins with antiviral activities. Sensing of dsRNA by oligoadenylate synthetases leads to production of 2′,5′-oligoadenylates, which activate host ribonuclease (RNase) L to degrade host and viral single-stranded (ss)RNA. Activation by protein kinase R (PKR) leads to dimerization and autophosphorylation followed by phosphorylation of eIF2α and inhibition of translation. Both RNase L and PKR activation lead to reduced virus replication, apoptosis, and inflammation (39). Thus, differential responses to these pathways are a potential explanation for the differences in the pathogenesis of the VOCs.

**Fig 4:**
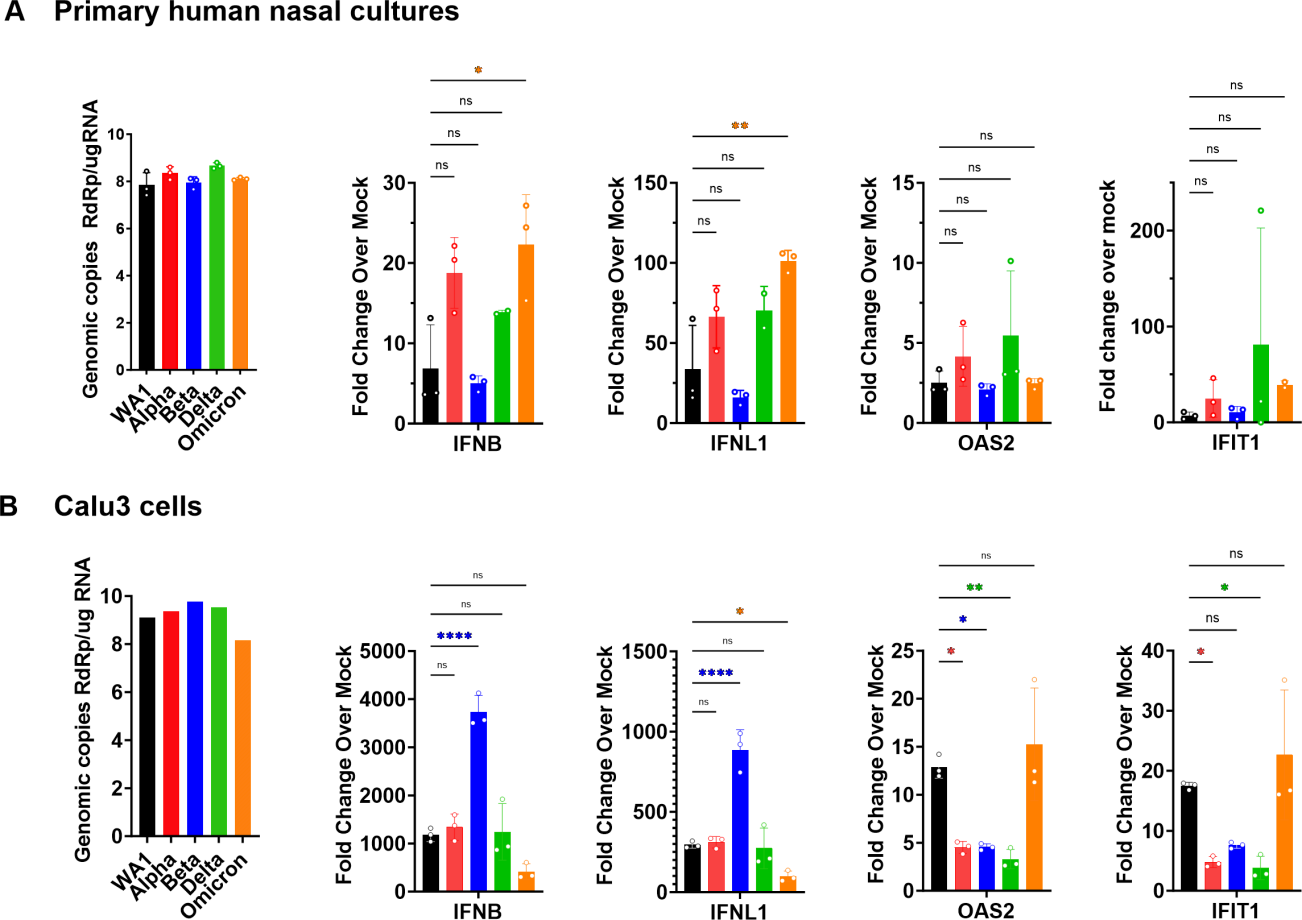
RT-qPCR of interferon and interferon-stimulated responses to SARS-CoV-2 infections. (A and B) Infections (MOI = 0.1) with indicated viruses were performed in (**A**) primary nasal cultures infected for 96 hpi and (**B**) Calu3 cells for 32 hpi. Cells were lysed for RNA extraction, and viral genomes were quantified by RT-qPCR with primers specific for SARS-CoV-2 RdRp, and human *IFNB*, *IFNL1*, *OAS2*, and *IFIT1*. Black bars indicate WA1, red for Alpha, blue for Beta, green for Delta, and orange for Omicron. For each cell type, graphs represent one of two independent experiments, each performed in biological triplicates. For primary nasal cultures, each experiment included a pool culture of three donors. Graphed values are mean with standard deviation, and statistics were performed with ordinary one-way ANOVA comparing VOCs versus WA1, adjusted *P*-values: *P < 0.01, **P < 0.001, ****P < 0.00001, ns, not significant.

To compare the induction of the interferon pathway among the SARS-CoV-2 viruses, RT-qPCR was used to quantify gene expression of type I IFN (*IFNB*), type III IFN (*IFNL1*), and ISGs, including 2′−5′-oligoadenylate synthetase 2 (*OAS2*) and interferon-induced protein with tetratricopeptide repeats 1 (*IFIT1*) in RNA from infected cells (Fig. 4). Host responses to infection were measured in both primary nasal cultures (Fig. 4A) and Calu3 cells (Fig. 4B) to understand if all viruses activate the same host response in both cell types, or if there is a cell type or a VOC-dependent response. To ensure productive virus replication, genome copy levels were quantified from infected cell lysates by performing RT-qPCR using primers to amplify nsp12 RNA-dependent RNA polymerase, RdRp, sequences. High genome copies of RdRp confirmed that all viruses reached high and productive replication in both cell types. The overall ratios of gene expressions do not correlate with virus genome copy levels, suggesting a reliable quantification of innate-immune responses induced in primary nasal cultures and Calu3 cells that are not skewed by viral RNA copy numbers.

We observed that while all viruses activated IFNB and IFNL1 expression above mock levels in primary nasal cultures, Omicron significantly induced these pathways (Fig. 4A). Consistent with these data, previous studies comparing IFN induction between Delta and Omicron infections in cell lines have also reported greater induction with Omicron (40, 41). However, we did not observe any differences by RT-qPCR for the ISGs, OAS2, and IFIT1, among the viruses (Fig. 4A). In contrast, in Calu3 cells, Beta generated significantly greater IFN responses than the other viruses. However, induction of ISGs was still the highest with Omicron (Fig. 4B). The discrepancies in gene induction between nasal cultures and Calu3 cells suggest cell-type-dependent host responses, and in both contexts, the infection was able to overcome cellular responses and lead to a productive replication.

**Fig 5:**
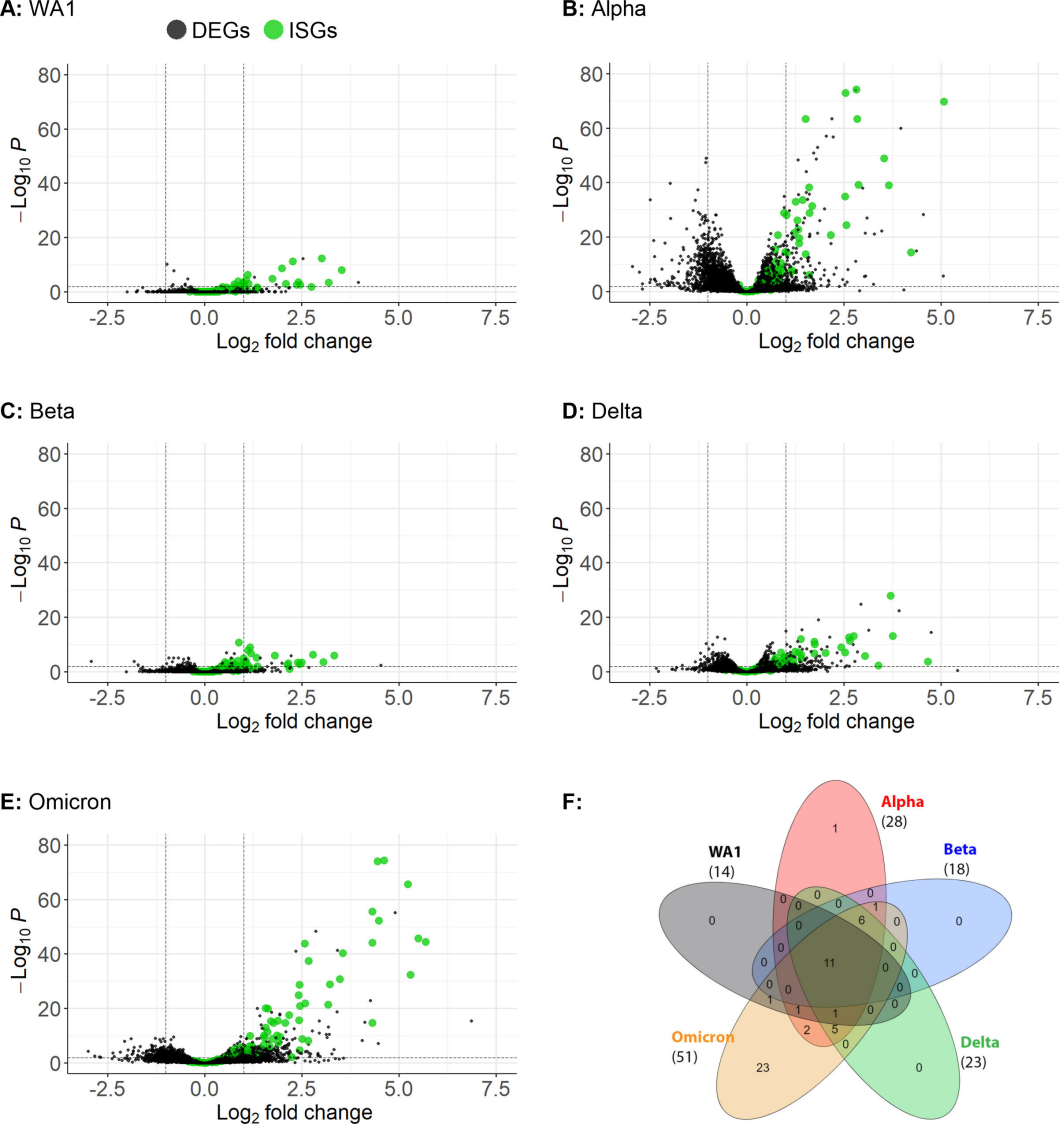
RNA-Seq of primary nasal cultures infected with SARS-CoV-2 WA1 and VOCs. (**A–E**) Primary nasal cultures pooled from four donors were infected with WA1 or VOCs at MOI = 0.1, and RNA was extracted at 96 hpi for RNA-Seq analysis. Each volcano plot represents significantly differentially expressing genes for the indicated infection compared to mock-infected primary nasal cultures. Black dots indicate DEGs, and green dots highlight interferon-stimulated genes. The number of graphed variables per condition is (**A**) 13,614, (**B**) 13,385, (**C**) 13,623, (**D**) 13,452, and (**E**) 13,515. (**F**) Venn diagram of signifiacnt ISGs, parathesis indicate the number of ISGs with a significance threshold of log2 fold change greater than 1 and *P*.adj value less than 0.01. A detailed list of this ISGs can be found in Table S1.

To understand the activation of IFN and ISGs on a broader scale, we sequenced RNA from lysates of primary nasal cultures infected with each of the viruses (Fig. 5). RNA-Seq data generated from infected and mock-infected cells were analyzed for differentially expressed genes (DEGs) that exceed the threshold of log2 fold change greater than 1 and *P*-adjusted value less than 0.01 for hits with high significance. Infection with WA1 induced the lowest number of DEGs, while infection with Omicron induced the highest number of DEGs (16 and 823, respectively). Infection with Alpha and Delta also resulted in a relatively high number of DEGs (475 and 202, respectively), while Beta was relatively lower (23 DEGs). All DEGs were processed for ISG gene ontology, which yielded a striking number of ISGs associated with Omicron infection (51 ISGs), followed by Alpha infection (28 ISGs) (Fig. 5B and E; Table S1). While infections with Delta (23 ISGs), Beta (18 ISGs), and WA1 (14 ISGs) also generated ISG responses, the number and amplitude of the upregulation were much lower than those induced by Omicron and Alpha infections.

In comparisons of specific ISGs, the levels of OAS2 and IFIT1 mRNA induction were similar among infections with WT and all VOCs by both RT-qPCR and RNA-Seq, suggesting common IFN signaling responses to the variants. However, a Venn diagram comparing highly significant ISGs shows that Omicron infection induces additional ISGs at higher levels than the other infections (Fig. 5F). This suggests that Omicron selectively induces certain ISGs. Overall, our data show that Omicron infection induces the strongest IFN and ISG responses.

### Double-stranded RNA-induced innate immune responses to SARS-CoV-2 variants

**Fig 6:**
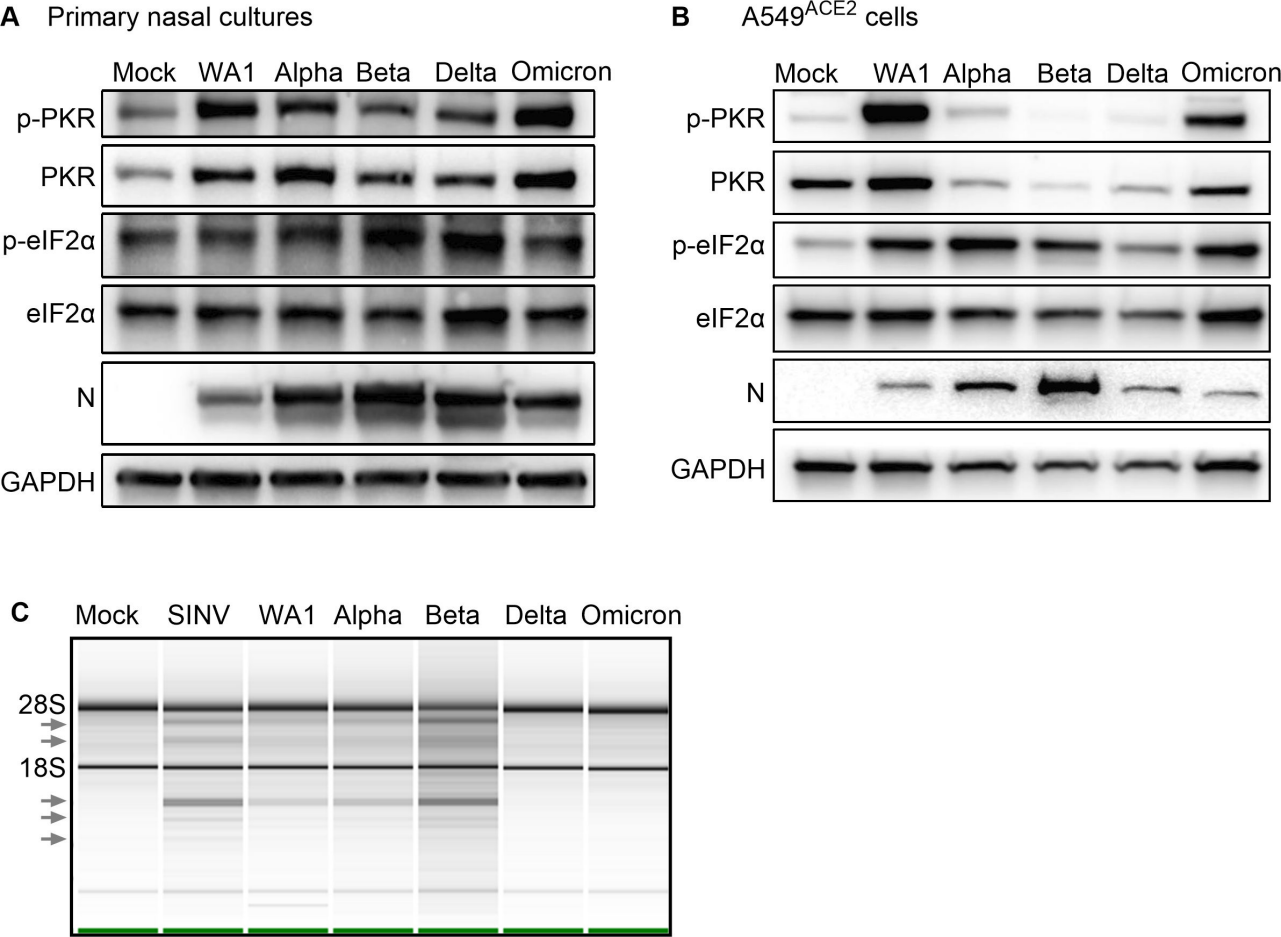
dsRNA-induced pathways during SARS-CoV-2 WA1 and VOCs infections. (**A**) Primary nasal cultures were infected at MOI = 0.1. Cells were lysed at 96 hpi, protein extracted, and analyzed by western blot for p-PKR, PKR, p-eIF2α, and eIF2α. The controls, GAPDH and SARS-CoV-2 nucleocapsid (N), are the same as Fig. 2A. (**B**) A549^ACE2^ cells were infected at MOI = 0.1 for 72 hpi. Cells were lysed, protein extracted, and analyzed by western blot for p-PKR, PKR, p-eIF2α, eIF2α, N, and GAPDH. (A and B) Images are representative of western blots from three independent infections. (**C**) Infections were performed in A549^ACE2^ cells at MOI = 0.1. At 48 hpi (SARS-CoV-2 strains) and 24 hpi (SINV), cells were lysed, RNA extracted, and analyzed on a Bioanalyzer. Arrows indicate bands of degraded RNAs. Image is representative of two independent experiments.

The activation of the PKR pathway during SARS-CoV-2 infection of primary nasal cultures and A549^ACE2^ cells was assessed by western blot (Fig. 6A and B). Phosphorylated-PKR (p-PKR) was detected in lysates of nasal cultures infected with all strains (Fig. 6A) and in A549 ^ACE2^ cells for WA1, Alpha, and Omicron (Fig. 6B), above the level of mock-infected cells, indicating that SARS-CoV-2 WA1 and VOCs activate the PKR pathway. It is worth noting that in primary nasal cultures, PKR, which is an ISG, was also detected above mock levels. Phosphorylated-eIF2α (p-eIF2α) was also detected during infection with all strains in A549 ^ACE2^ cells. However, the level of p-eIF2α over mock-infected cells was variable in nasal cultures, a reproducible observation for the nasal culture model, which may be due to the low percentage of infected cells and that eIF2α level is not IFN induced while PKR level, as well as phosphorylation of PKR, is IFN dependent. Together, these results suggest that all SARS-CoV-2 infections in primary nasal cultures and A549^ACE2^ cells induce the PKR and eIF2α pathway (Fig. 6A and B).

The activation of the RNase L pathway can be inferred by assessing rRNA degradation using a Bioanalyzer (Fig. 6C). Due to consistently undetectable levels of degradation of rRNA bands in lysates from primary nasal cultures, possibly due to a low percentage of infected cells as above for p-eIF2α levels, we assessed lysates from infected A549^ACE2^ cells for rRNA degradation. Compared to the mock-infected cell, the positive control SINV infection was associated with the accumulation of rRNA degradation products (arrows), as expected. Among the SARS-CoV-2 viruses, WA1, Alpha, and Beta generated rRNA degradation products. The reduced intensities of rRNA degradation in Delta and Omicron infection (Fig. 6C) could be due to delayed progression of infection, as indicated by lower nucleocapsid (N) levels (Fig. 6B) and titer (Fig. S2C) in A549^ACE2^ cells, rather than a muted response by the RNase L pathway.

### Damage to upper respiratory cells associated with Delta infection

**Fig 7:**
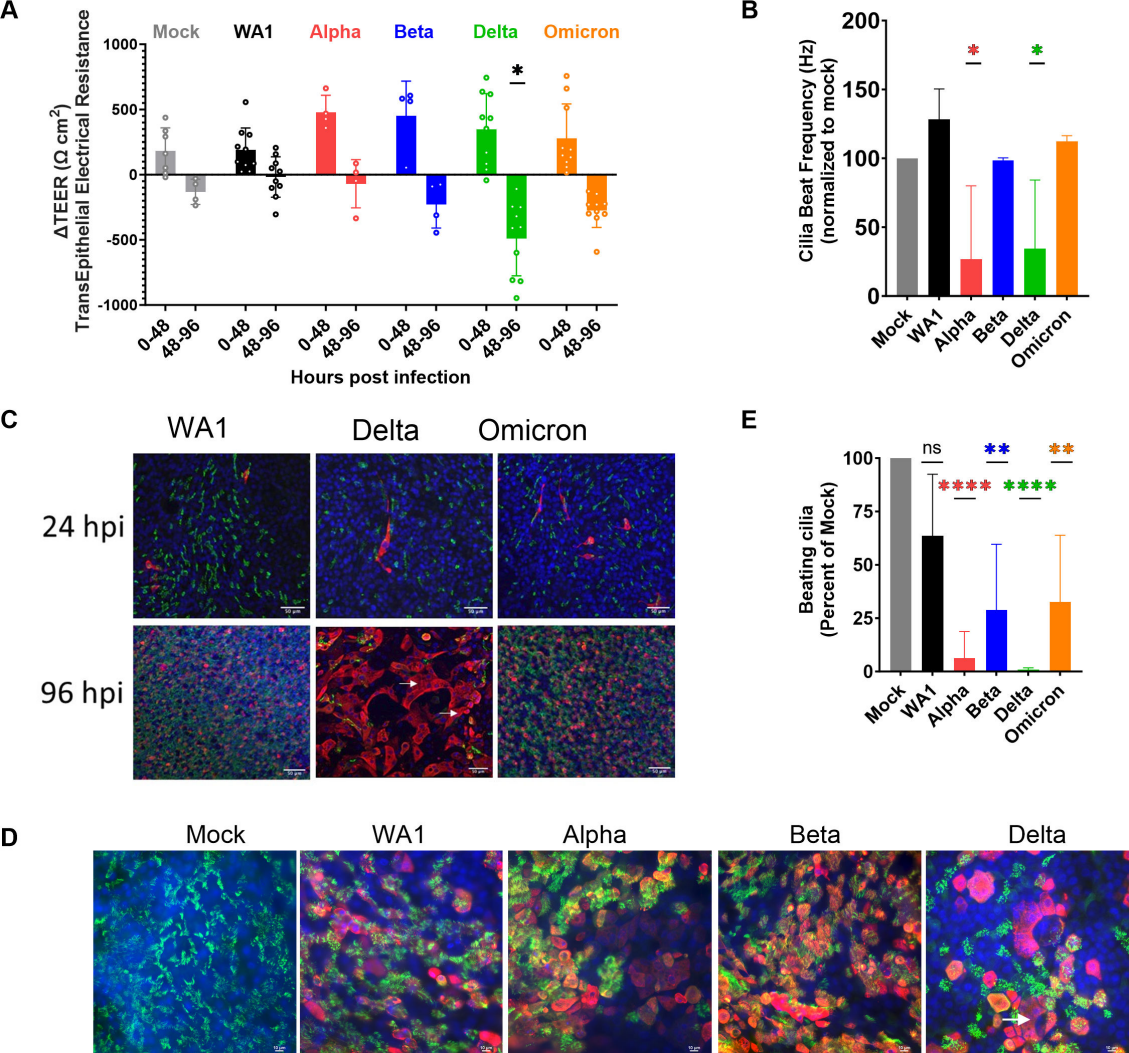
Infection with SARS-CoV-2 VOCs damages primary nasal cultures. Primary nasal epithelial cultures were infected with SARS-CoV-2 viruses at MOI = 0.1 and processed at 96 hpi for various assays. (**A**) Transepithelial electrical resistance was quantified during SARS-CoV-2 infection of nasal cultures at 0, 48, and 96 hpi. Values represent the difference in TEER (∆TEER) during the indicated time span and averaged from three independent experiments. Graphed values are mean with standard deviation, and statistics were performed with ordinary two-way ANOVA comparing VOCs versus WA1 within a time span, multiple comparison adjusted *P*-value: *P < 0.01. (**B**) Ciliary beat frequency was measured in mock and infected nasal cultures at 96 hpi. (**C and D**) Mock and infected nasal cultures from separate donors were fixed at 96 hpi for immunofluorescence and confocal imaging. Antibodies were used to label DAPI (blue), β-tubulin (green), and SARS-CoV-2 nucleocapsid (red). Arrows indicate syncytia-like clusters. Scale bars indicate 50 (**C**) and 10 um (**D**). Images are representative of infections in multiple donors. (**E**) Real-time videos of mock and infected nasal cultures at 96 hpi were quantified for the area of actively beating cilia. (**B and E**) Graphed values represent mean with standard deviation, and statistics were performed with one-way ANOVA using Dunnett’s multiple comparisons test for all infected conditions against mock/uninfected, with adjusted *P*-values: *P < 0.05, **P < 0.005, ****P < 0.00005, ns, not significant.

Infection by SARS-CoV-2 can last for prolonged periods, raising the question of possible damage to infected tissue. To compare the damage caused by the different variants, we measured the transepithelial electrical resistance (TEER), which measures electrical resistance across the cell membranes that is maintained by intact cell-to-cell junctions. As the nasal cultures become more confluent and differentiate, the TEER values rise; however, any compromise to cell-to-cell barrier integrity leads to loss of ion transport across the membrane which can be measured as a reduction of TEER value. We observed that while all VOCs display an initial increase in TEER (0–48 hpi), there is a significant drop in TEER during late infection (48–96 hpi) (Fig. 7A). The most dramatic and significant loss of TEER was observed during Delta infection, suggesting that Delta induces significantly more damage to cell-to-cell barrier integrity than other variants.

The nasal cultures include epithelial cells with projections of cilia on the apical surface, which facilitate the transport and clearance of mucus that is generated in the nasal cavity. Beat frequency can be measured to assay the activity and function of cilia as well as overall health of nasal cultures. Compared to uninfected mock cells, nasal cultures infected with Alpha and Delta displayed a notable 75% loss in ciliary beating frequency (Fig. 7B). This suggests that SARS-CoV-2 infection compromises ciliary beating function in the nasal cell cultures, with different potency among variants.

Confocal microscopy was used to visualize the spread of infection and possible cell-to-cell fusion. During early infection (24 hpi), we observed that infected cells colocalized with the ciliary marker β-tubulin, confirming that SARS-CoV-2 infects ciliated cells (Fig. 7C) (16). Additionally, only a fraction (<5%) of the ciliated cells was infected at this early time point. During late infection (96 hpi), we observed a vast spread of infection throughout the nasal cultures. Notably, Delta-infected nasal cultures showed some small syncytia-like clusters (Fig. 7C, arrow), although not as pronounced as observed in lower respiratory cell lines (Fig. 2C). At the least, these cultures are indicative of infectious centers with a shared cytoplasm (42). The loss of the cytoskeletal marker, phalloidin, between cells in this cluster is further evidence of the formation of syncytia-like clusters (Fig. S3C). At higher magnification, we observed that during later infection (96 hpi), Delta-infected cells were negative for staining for β-tubulin, suggesting the deciliation of the infected cells (Fig. 7D). Using live microscopy and point analysis, we also observed a reduction in the area where beating cilia could be detected (Fig. 7E), further documenting deciliation. Together, these results suggest that among the SARS-CoV-2 viruses, Delta VOC was the most cytopathic among WA1 and the other VOCs in human nasal epithelia.

### Population surveillance for SARS-CoV-2 reveals Delta and Omicron as fastest replicating

**Fig 8:**
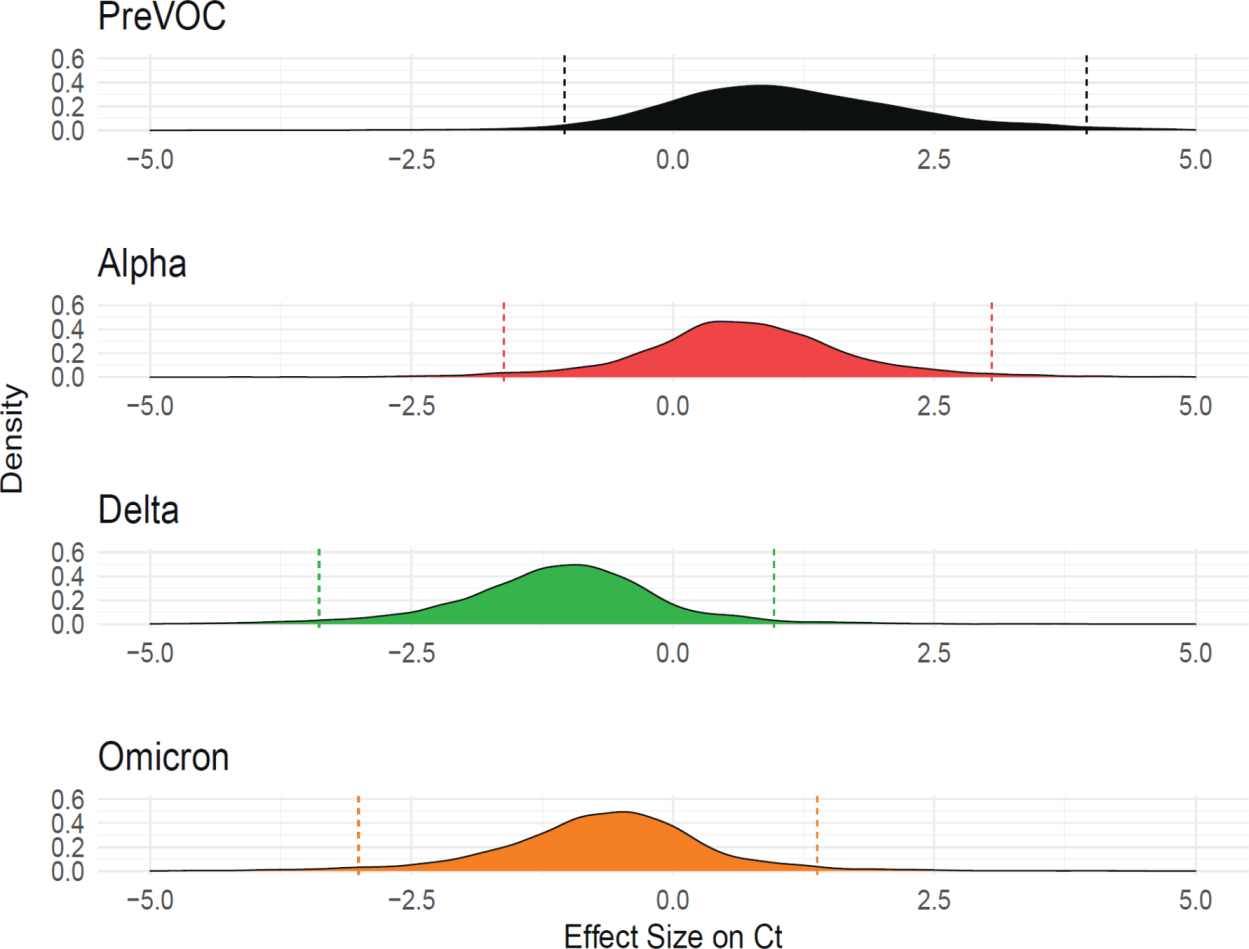
Delaware Valley SARS-CoV-2 surveillance data. Effect size of variants on clinical qPCR assay Ct (compared to pre-VOC), adjusted for time during wave, specimen type, and qPCR machine as captured using a Bayesian regression model. The *X*-axis shows the marginal effect on Ct for each variant compared to pre-VOC. Note that one cycle change in Ct is roughly equivalent to a twofold increase in RNA abundance under ideal PCR conditions. The *Y*-axis shows the posterior probability density generated from the Bayesian regression model.

Given our goal of understanding molecular correlates of differences among variants, and the results described above, we investigated whether infections with different SARS-CoV-2 variants were associated with higher viral RNA levels as reported by RT-qPCR assays on clinical specimens. Samples were obtained from a program that paired viral whole-genome sequencing with a collection of clinical metadata so that relative viral RNA levels were linked to the variant calls (43). Samples were analyzed from 2,722 infected participants from the Delaware Valley region. Our analysis pools cycle of threshold (Ct) values from different analytical technologies that were in place based on the location of sample collection and the stage of the epidemic (Fig. S1B). Due to the low prevalence of Beta infection in the Delaware Valley region, our data do not include this VOC. Data from several sampling and analysis pipelines were combined, so a Bayesian analytical framework was used to control confounders and assess the significance of differences among variants (Fig. 8). Relative abundance was quantified as cycle of threshold in the qPCR so that lower values indicate higher abundances. It is worth noting that some variants were associated with overwhelmed healthcare systems which might have resulted in longer wait times for testing, which could influence Ct values for some data points. These potentially longer durations between symptom onset and tests could result in reduced measured viral RNA levels. However, the need for this type of correction is unknown due to the lack of data for this time interval. This lack of data also prohibits correction if needed.

We found that Delta specimens had Ct values that were estimated to be on average 1.77 Ct lower than Alpha [95% credible interval (CrI): 0.70, 2.89] and 0.43 Ct lower than Omicron (95% CrI: 0.15, 0.72). Omicron was estimated to have a Ct 1.35 cycles lower than Alpha (95% CrI: 0.28, 2.42). Delta was consistently found to have a lower Ct and thus higher numbers of RNA copies in infected human participants, followed by Omicron and Alpha. This finding is consistent with published studies of patient samples (44–47) and matches our findings that Delta and Omicron VOCs are most efficient at replicating in primary nasal cultures (Fig. 1).

## DISCUSSION

The COVID-19 pandemic has been marked with emergence of SARS-CoV-2 variants including Alpha, Beta, Delta, and Omicron, which were designated as variants of concern due to their heightened risk to the population. Infections with different VOCs have shown notable differences in patient health outcomes (48), but understanding of the differences in viral biology accounting for these differences is incomplete. A comparative mechanistic analysis of SARS-CoV-2 variants is necessary to delineate the virus-host biology driving pathogenesis and to help predict the pathogenicity of future variants. Our findings parallel and provide mechanistic insight into retrospective human studies (6, 48) which showed that infections with Delta are most pathogenic, while Omicron is less pathogenic and instead selected for better replication (6).

Our findings focus attention on the differences in infections among SARS-CoV-2 viruses in primary nasal cultures and cell lines that facilitate technical aspects of some assays that are not feasible in primary nasal cultures. The cell lines used include A549 and Calu3, which allow us to compare infection in human lower respiratory tract-derived cells. However, a caveat of such comparison is the effects of primary cultures versus cell lines derived from carcinomas to the observations. A strength of our study lies in the analysis of infection in patient-derived primary nasal cultures which are the primary site of infection and model the upper respiratory tract (38). Unlike cell lines or animal models, our findings with the primary nasal culture model parallel human epidemiological studies in the comparison of infections by the different variants, in that, in both models, Delta and Omicron were selected for efficient replication compared to other VOCs.

A consistent observation throughout our studies is that Delta infections are most damaging in both upper and lower respiratory models. This could be attributed to the polybasic furin cleavage site on the Delta spike protein, most basic among the VOCs, which can recruit proteases to facilitate spike cleavage and subsequent spread of infection. Despite sharing an identical furin cleavage site, Alpha and Omicron infections generate different levels of cleaved spike, cell-to-cell spread of virus, and cellular damage. Thus, our results indicate that factors in addition to the cleavage site play a role in these processes. Sequence and structure-based bioinformatics studies have proposed that mutations in Omicron-encoding substitutions at the S1/S2 site may help evade recognition by proteases and prevent syncytia formation (49), which may explain our observation of less than expected levels of spike cleavage and propensity toward endosomal entry. However, more recent studies using nasal organoids have reported increased syncytia formation with the Omicron BA.5 subvariant compared to the first Omicron BA.1 variant used in our study (50, 51).

Differences in innate immune responses to infections by different VOCs have been studied extensively. We have shown that Omicron infection induces greater *IFNB* and *IFNL* gene response in primary nasal cultures compared to the ancestral strain. However, in Calu3 cells, Beta infection elicited a stronger IFN response that surpassed that of infection with other VOCs. These variations may be explained by several factors. First, different cell types have different capacities for generating IFN responses. For example, it has been shown that nasal cultures have elevated basal levels of interferon gene expression, and thus, induction may appear to be blunted (38). Second, the level of innate responses may correlate with viral load in cells, and different VOCs replicate to different relative titers in different cell types.

Clinical studies of COVID-19 patient outcomes have proposed that patients with an early and robust IFN response were more likely to have mild-to-moderate disease, while patients with a delayed or blunted response were more likely to succumb to severe COVID (52, 53). The high induction of IFN and ISGs with Omicron infections in our model would suggest that this induction may contribute to the milder disease associated with Omicron (5, 6). Recent studies also observed greater ISG induction with Omicron (54) and reduced IFN antagonism by Omicron as contributing to the milder disease (40). However, there are many confounding factors that warrant further experimentation. For example, adaptive immunity from an earlier infection or a vaccine may influence the severity and general outcome of later infections at the population level.

Primary human nasal epithelia cultures grown at an ALI are a powerful model to recapitulate coronavirus infections of the upper respiratory tract (38, 55). Previous studies have validated SARS-CoV-2 infections in such models (16, 56, 57). Here, we were able to characterize specific forms of cellular damage in ciliated cells by different VOCs. Immunofluorescence analysis shows that Delta is the most cytopathic VOC, followed by Alpha and Omicron. The severity of cytopathic effect suggests cell death, although we have not determined the specific type(s) of cell death. This order was also observed with levels of spike cleavage, suggesting that in nasal cultures Delta may spread by cell-to-cell fusion more than other VOCs. Analysis of TEER also identified Delta as the most damaging to cell barrier integrity, followed by Omicron. However, further analysis suggests that Delta and Alpha caused the greatest loss of ciliary beating function. Therefore, while Delta infection compromises both functions, Omicron is detrimental to cell barrier integrity more than ciliary beating, and Alpha infection diminishes ciliary beating but not cell barrier integrity. This contrast highlights how the variants differentially affect mechanisms crucial for nasal epithelial functions at the cellular level that may correlate with variant-specific patient symptoms.

In primary nasal cultures, Delta and Omicron replicated to higher titers faster, while WA1 produced the lowest titer. In contrast, in the Calu3 cell line, replication of the earlier VOCs, such as Alpha and Beta, reached higher titers earlier. These results suggest that SARS-CoV-2 is evolving for efficient replication in the upper respiratory tract. This observation concurs with clinical studies that report Omicron infections induce more upper respiratory symptoms with reduced pathogenic manifestation (58, 59). These observations suggest future variants may continue to be selected for upper respiratory infections and a general decrease in pathogenicity.

While the use of primary nasal epithelial culture model provides many advantages to our study, there are some caveats to be aware of. The heterogeneity of cell types in the nasal epithelium compromises pathway and mechanistic analysis. While cell lines are used to circumvent these technical problems, this introduces confounding factors that are driven by cell line specific biology. The primary nasal cultures also lack an intact immune system that would be present in the human.

The currently circulating SARS-CoV-2 virus is likely to continue to evolve. The findings in this study provide a comprehensive comparison of VOCs to date in cell culture, clarifying important differences in virus-host biology among SARS-CoV-2 variants affecting pathogenesis. These findings can be applied to understand and predict the replication, spread, and immunogenicity of future variants.

## MATERIALS AND METHODS

### Viruses, replication curves, and plaque assays

The following viruses were obtained from BEI resources, WA1/USA-WA1/2020 strain NR-52281 and Alpha NR-54000. Delta and Omicron were isolated from patient samples. All viruses were grown in VeroE6^TMPRSS2^ cells, and titers were quantified by plaque assay on VeroE6 monolayer overlayed with 0.1% agarose and stained with 10% crystal violet (38). All viruses were sequence verified using the POLAR protocol using an Illumina NextSeq instrument with a 74 × 74 paired-end sequencing on a 150-cycle MID output cartridge (60). Viral isolate sequences are available at GenBank using the following accession: WA1: PP237008, Alpha: PP237009, Beta: PP237012, Delta: PP237010, and Omicron: PP237011.

Infections were performed at MOI = 0.1, unless otherwise specified, in serum-free DMEM for 1 hour, followed by refeeding of cellular media for the duration of the experiment. For virus growth curve experiments, virus supernatant was collected at noted hours post infection and quantified by plaque assay. For intracellular virus, cells were collected in DMEM and subjected to three freeze-thaw cycles to release the intracellular virus.

### Cell lines

VeroE6^TMPRSS2^ (African green monkey kidney) cells were maintained in DMEM (Gibco Cat. No. 11965) with 10% fetal bovine serum (FBS), 100 U/mL penicillin, 100 ug/mL streptomycin, 50 ug/mL gentamicin, 1 mM sodium pyruvate, and 10 mM HEPES. Human A549^ACE2^ cells were cultured in PRMI 1640 (Gibco Cat. No. 11875), 10% FBS, 100 U/mL of penicillin, and 100 ug/mL streptomycin. Human Calu3 cells (clone HTB-55) were maintained in MEM media with 10% FBS.

### Genomes/PFU

Calu3 cells were infected with WA1 and VOCs at MOI 0.1, and at 24 hpi, virus supernatant was collected. Infectious virus was quantified by plaque assay. Genomes were measured by extracting RNA from the supernatant and performing RT-qPCR with primers for SARS-CoV-2 nsp12 (RdRp) sequences.

### RNA extraction

Cells were lysed at indicated hours post infection in Buffer RLT (Qiagen Cat. No. 79216) followed by extraction of RNA using the RNeasy Plus Mini Kit (Qiagen Cat. No. 74004). Cell-free supernatant was lysed with AVL buffer (Qiagen Cat. No 19073), and RNA was extracted with QIAmp Viral RNA Mini Kit (Qiagen 52904).

### RT-qPCR

The protocol for RT-qPCR was previously described and briefly outlined here (61). RNA was reverse transcribed into cDNA with a high capacity cDNA Reverse Transcriptase Kit (Applied Biosystems). Target cDNA was amplified using specific primers, iQ SYBR Green Supermix (Bio-Rad) and QuantStudio 3 PCR system (Thermo Fisher). Host gene expression displayed as fold change over mock-infected samples was generated by first normalizing cycle threshold (Ct) values to 18S rRNA to generate ΔCt values (ΔCt = Ct gene of interest − Ct 18S rRNA). Next, Δ (ΔCt) values were determined by subtracting the mock-infected ΔCt values from the virus-infected samples. Technical triplicates were averaged, and means displayed using the equation 2^−Δ (ΔCt)^. Graphed values are the mean of biological triplicates of each condition and technical triplicates of each sample. Host gene expression was quantified with the following primers (forward sequence/ reverse sequence): IFNB (GTCAGAGTGGAAATCCTAAG/ CAGCATCTGCTGGTTGAAG), IFNL1 (CGCCTTGGAAGAGTCACTCA/ GAAGCCTCAGGTCCCAATTC), OAS2 (TTCTGCCTGCACCACTCTTCACGAC/ GCCAGTCTTCAGAGCTGTGCCTTTG), IFIT1 (TGGTGACCTGGGGCAACTTT/ AGGCCTTGGCCCGTTCATAA), and 18S rRNA (TTCGATGGTAGTCGCTGTGC/ CTGCTGCCTTCCTTGAATGTGGTA). Virus genomes were quantified in reference to a standard curve, with primers for SARS-CoV-2 genomic nsp12/RdRp (GGTAACTGGTATGATTTCG/ CTGGTCAAGGTTAATATAGG).

### RNA sequencing

Nasal epithelial cells from four donors were pooled together into air-liquid interface cultures. Mock infections or infections with the indicated viruses were performed in triplicate at MOI = 0.1. Cells were lysed at 96 hpi using RLT Plus buffer, and total RNA was extracted using Qiagen RNeasy Plus Mini kit (Cat. No. 74004). Samples were sent to Azenta Life Sciences for RNA sequencing with Illumina HiSeq PE 2 × 150. Read quality was assessed using FastQC v0.11.2 (62). Raw-sequencing reads from each sample were quality and adapter trimmed using BBDuk 38.73 (63, 64). The reads were then mapped to the human genome (hg38 with Ensembl v98 annotation) using Salmon v0.13.1 (65). Differential expression between mock and infected experimental conditions was analyzed using the raw gene count files by DESeq2 v1.22.1 (66). Volcano plots were generated using EnhancedVolcano v1.14.0 (67), with highlighted interferon-stimulated genes being selected from the Molecular Signatures Database HALLMARK_INTERFERON_ALPHA_RESPONSE gene list (68). Venn diagram was generated using the InteractiVenn web tool (69).

### Graphical visualization, quantification, and statistics

Graphs were generated using Prism software. Statistics were also performed with Prism software with specific tests stated with each experiment. Plaque area quantification, syncytia area quantification, and western blot quantification were performed with ImageJ software.

### Protease treatments

Nafamostat (20 uM) was used for protease inhibition (70). Calu3 cells were pre-treated for 2 hours with appropriate concentrations of drug in the cell media. Infections were performed at MOI = 0.1 for 1 hour, after which inoculum was replaced with Calu3 media plus drug for one additional hour of post-treatment. After this, cell media were replaced with drug-free media for the remainder of the experiment. For positive control, drug concentration was maintained in the media for 4 hours of post-treatment. For negative control, no drug was included in the media, and DMSO media (dimethyl sulfoxide, Thermo Scientific Cat. No. J66650) was used instead. Supernatants were collected at 16 hpi for quantification by plaque assay. Percent inhibition was calculated as fraction of virus titer after drug treatment over virus titer without drug treatment.

### RNase L degradation assay

RNA integrity was analyzed on a chip with Aligent 2100 Bioanalyzer using the Aligent 196 RNA 6000 Nano Kit (Cat #: 5067–197 1511).

### Western immunoblot

Cells were washed in PBS and harvested in lysis buffer (1% NP-40, 2 mM EDTA, 10% glycerol, 150 mM NaCl, 50 mM Tris-HCl), protease inhibitor (Roche complete mini EDTA-free protease inhibitor), and phosphatase inhibitor (Roche PhosStop easy pack). After 20 minutes of lysis on ice, samples were centrifuged to remove cell debris. Lysates were denatured at 95°C for 5 minutes and stored for analysis. Protein lysates were separated on 5%–15% SDS-PAGE gradient gel and transferred onto a PVDF membrane. Membrane blots were blocked with 5% nonfat milk or 5% BSA and probed with appropriate primary antibodies overnight at 4°C and secondary antibodies for 2 hours at room temperature. Blots were exposed with chemiluminescent substrate (Thermo Scientific Cat. No. 34095 or 34080). Blots were stripped (Thermo Scientific Cat. No. 21059) and reblotted as needed. Primary antiobdies used in this assay include SARS-CoV-2 S2 (anti-mouse, 1:3000, Biolegend Cat. No. 943202), SARS-CoV-2 Nucleocapsid (anti-rabbit, 1:5000, ProSci Cat. No. 9099), GAPDH (anti-rabbit, 1:2000, Cell Signaling Technology Cat. No. 2118S), p-PKR (anti-rabbit, 1:1000, Abcam Cat. No. 32036), PKR (anti-rabbit, 1:1000, Cell Signaling Technology Cat. No. 12297S), p-eIF2α (anti-rabbit, 1:1000, Cell Signalining Technology Cat. No. 9721S), eIF2α (anti-rabbit, 1:1000, Cell Signalining Technology Cat. No. 9722S). HRP-conjugated secondary antibodies include goat anti-rabbit IgG and goat anti-mouse IgG (1:3000, Cell Signaling Technologies Cat. No. 7074S and 7076S).

### Primary human nasal cultures

Sinonasal cells were obtained from patient donors with informed consent, per protocol approved by the University of Pennsylvania Institutional Review Board (protocol #800614). A detailed protocol was described previously (38, 71). Briefly, specimens were dissociated and grown to 80% confluence in in PneumaCult-ALI Medium (STEMCELL Technologies 05001) supplemented with heparin (STEMCELL Technologies 07980) and hydrocortisone. Once cells reach confluency in 0.4 µM pore transwell inserts, the apical growth media are removed, and the basal differentiation media are replaced every 3–4 days for a minimum of 4 weeks. Prior to infection, epithelial morphology and cilia beating are confirmed visually by microscopy.

### Transepithelial electrical resistance

TEER measurements were obtained with the EVOM apparatus, in PBS supplemented with calcium and magnesium. TEER measurements were obtained for all nasal ALI culture transwells pre-infection (0 hpi) and post-infection (48 and 96 hpi). ∆TEER is the difference in TEER from 0 to 48 hpi, and 48 to 96 hpi.

### Cilia beat frequency and beating cilia

Live microscopy movies of nasal cultures were obtained with a 20× objective on a brightfield microscope. Movie segments were analyzed on SAVA system to obtain cilia beat frequency (72). Beating cilia was obtained with single-point analysis. Graphed values are an average of three nasal cultures per condition and four regions of interest per culture.

### Immunofluorescence

A549^ACE2^ cells were seeded on coverslips and infected at confluency at MOI = 0.1. At 24 hpi, samples were fixed with 4% paraformaldehyde for 30 minutes, followed by permeabilization with 0.5% trypsin for 10 minutes. Samples were blocked with 1% BSA, followed by incubation with primary antibodies for 2 hours at room temperature and secondary antibodies for 1 hour at room temperature. Antibodies used include DAPI (1:1000, Invotrogen Cat. No. S36939), SARS-CoV-2 Nucleocapsid (anti-mouse, 1:500, gift from Dr. Tony Schountz, Colorado State University, Fort Collins, CO, USA), SARS-CoV-2 Nucleocapsid (anti-rabbit, 1:5000, Gentex Cat. No. GTX135357), Type IV β-tubulin (anti-rabbit, 1:1000, Abcam Cat. No. ab179509), Phalloidin-iFlour-647 (1:10000, Abcam Cat. No. ab176759), Alexa-Fluor-488 (1:1000, Thermo Scientific Cat. No. A11011 for anti-mouse, ab150077 for anti-rabbit) and Alexa-Fluor-546 (1:1000, Thermo Scientific Cat. No. AA11003 for anti-mouse, A10040 for anti-rabbit). Cell-line images were obtained with a 60× objective on a Nikon Ti-8 brightfield microscope. Confocal images of nasal cultures were captured on a Olympus Fluoview 113 System (Z-axis step 0.5μm, sequential scanning) and displayed as overlay projections.

### Ct analysis of Delaware Valley surveillance

Samples were collected from the Delaware Valley under University of Pennsylvania IRB protocol no. 823392, CDC BAA 200–2021-10986 and CDC 75D30121C11102/000HCVL1-2021-55232. RNA samples were analyzed by whole-genome sequencing as described previously (43, 73). Ct values were obtained from patient records. Clinical viral RNA level measured by cycle threshold was predicted using a Bayesian regression model as implemented in BRMS using a thin plate spline for time since variant first detected and random effect of qPCR machine, specimen type, and variant of sample.


$$Ct_{i}∼s\left(t_{i}\right)+\lambda_{i}+\alpha_{v}+\beta_{m}$$

where $Ct_{i}$ is an estimate of a sample’s Ct value. $s\left(t_{i}\right)$ is a thin plate regression spline calculated to account for variations over time for all variant’s introduction. $\lambda_{i}$ is the effect on sample location on either upper or lower respiratory tract. $\alpha_{v}$ is the effect for a given variant, and $\beta_{m}$ is the effect for a given qPCR protocol.

A total of 2,722 SARS-CoV-2-positive participants had clinical Ct data collected and were sequenced through whole-genome sequencing from 15 February 2021 to 18 July 2022. The supplemental table contains metadata and sequence accessions used in this analysis. One of six qPCR protocols was used (Cepheid GeneXpert, Cobas8800, Cobas Liat, DiaSorin MDX, Saliva COVID, or ThermoFisher Amplitude). One of five variant categories was assigned from whole-genome sequencing (Alpha, Delta, Omicron, other variant, or pre-variant of concern).

## Data Availability

Sequence data for viral isolates used in this work can be accessed through GenBank using the following accessions: PP237008-PP237012. Raw and processed RNA-seq data discussed in this publication have been deposited in NCBI's Gene Expression Omnibus and are accessible through GEO Series accession number GSE250155 (74).

## SUPPLEMENTAL MATERIAL

Supplemental Figures and Table mbio.03129-23-s0001.pdf Figures S1, S2, and S3 and Table S1.

ASM does not own the copyrights to Supplemental Material that may be linked to, or accessed through, an article. The authors have granted ASM a non-exclusive, world-wide license to publish the Supplemental Material files. Please contact the corresponding author directly for reuse.
