# Supplementary material for: Comparison of SARS-CoV-2 variants of concern in primary human nasal cultures demonstrates Delta as most cytopathic and Omicron as fastest replicating
Source: mBio. 2024 Mar 13;15(4):e03129-23. doi: 10.1128/mbio.03129-23 (PMC11005367; doi:10.1128/mbio.03129-23)
Supplement: Supplemental Figures and Table — Figures S1, S2, and S3 and Table S1. [file mbio.03129-23-s0001.pdf]

Supplemental Figure 1: SARS-CoV-2 infections in cell lines.

(A) SARS-CoV-2 stocks were harvested from VeroE6^{TMPRSS2} cells after 2 passages and RNA was collected from infected cells and sequenced. Viral sequences were aligned against the WA1 genome sequence for reference. Amino acid substitutions are indicated with colored circles, black for WA1, red for Alpha, blue for Beta, green for Delta and orange for Omicron. The fraction of sequences with indicated mutation is denoted as pie slice within the circle. 270 substitutions are represented across the 4 variants when compared to the reference wildtype. 240 of these substitutions are dominated by 95% or more with a single base call. The remaining 30 substitutions have a mixture of base calls in each position with no call more than 95% dominant. Sequence data for viral isolates used in this work can be accessed through GenBank using the following accessions: PP237008-PP237012. (B) Different analytical technologies and PCR protocols used to measure the graphed Ct values throughout the endemic.

Supplemental Figure 2: SARS-CoV-2 infections in lower respiratory cell lines

(A) VeroE6^{TMPRSS2} cells were infected at MOI=1 and lysed for protein at 48 hpi. Proteins were analyzed by western blot and probed with antibodies against nucleocapsid (N) and spike (S2). Band intensities from 3 independent western blots were quantified to measure the fraction of cleaved spike protein. The percent of cleaved spike was calculated as the fraction of cleaved spike (S2) over the sum of full length (FL) and cleaved (S2) spike. (B) Calu3 cells were infected with SARS-CoV-2 viruses (MOI=0.1) and RNA was collected at 24 hpi. RT-qPCR was performed with primers specific for SARS-CoV-2 nsp12 RdRp sequence to quantify virus genomes. (C) A549^{ACE2} cells were infected (MOI=0.1) and the supernatant was collected at indicated time points to quantify infectious virus by plaque assay. Graphed values represent mean with standard deviation, and statistics were performed with ordinary two-way ANOVA with multiple comparisons for VOCs versus WA1 within a time point, adjusted P-values: *P<0.05, **P<0.005, ***P<0.0005.

Supplemental Figure 3: WA1 and VOC infections in nasal cultures

(A) Nasal cultures (pooled from 4 donor cells) were infected (MOI =0.1) and incubated at 33°C or 37°C. Apically shed virus was collected at the indicated time points and infectious virus was quantified by plaque assay. Growth curves at 33°C are indicated with dashed lines, and 37°C with solid lines. Graphed values represent the mean with standard deviation and statistics were performed with ordinary two-way ANOVA with multiple comparisons for VOCs versus WA1 at the two temperatures within a time point. Values did not reach statistical significance. (B) Nasal cultures were infected at MOI=0.1; at 96 hpi samples were fixed and processed by immunofluorescence for DAPI (blue), β-tubulin (green), and SARS-CoV-2 nucleocapsid (red). (C) Infected and mock cultures (also depicted in Fig 7D) were fixed for immunofluorescence and confocal imaging with antibodies to label N (red) and phalloidin (white). The arrow indicates syncytia-like clusters. Images are representative of infections in multiple donor nasal cultures.

Supplemental Table 1: Significantly enriched interferon stimulated genes.

Differentially expressed genes from RNAseq of primary nasal cultures infected with WA1 or VOCs (Fig 5) were analyzed for interferon stimulated genes. ISGs that are differentially expressed in infected cells compared to uninfected cells and exceed a high significance threshold of Log2 fold change greater than 1 and adjusted P. value less than 0.01 are listed for each infection.

Fig S1

A

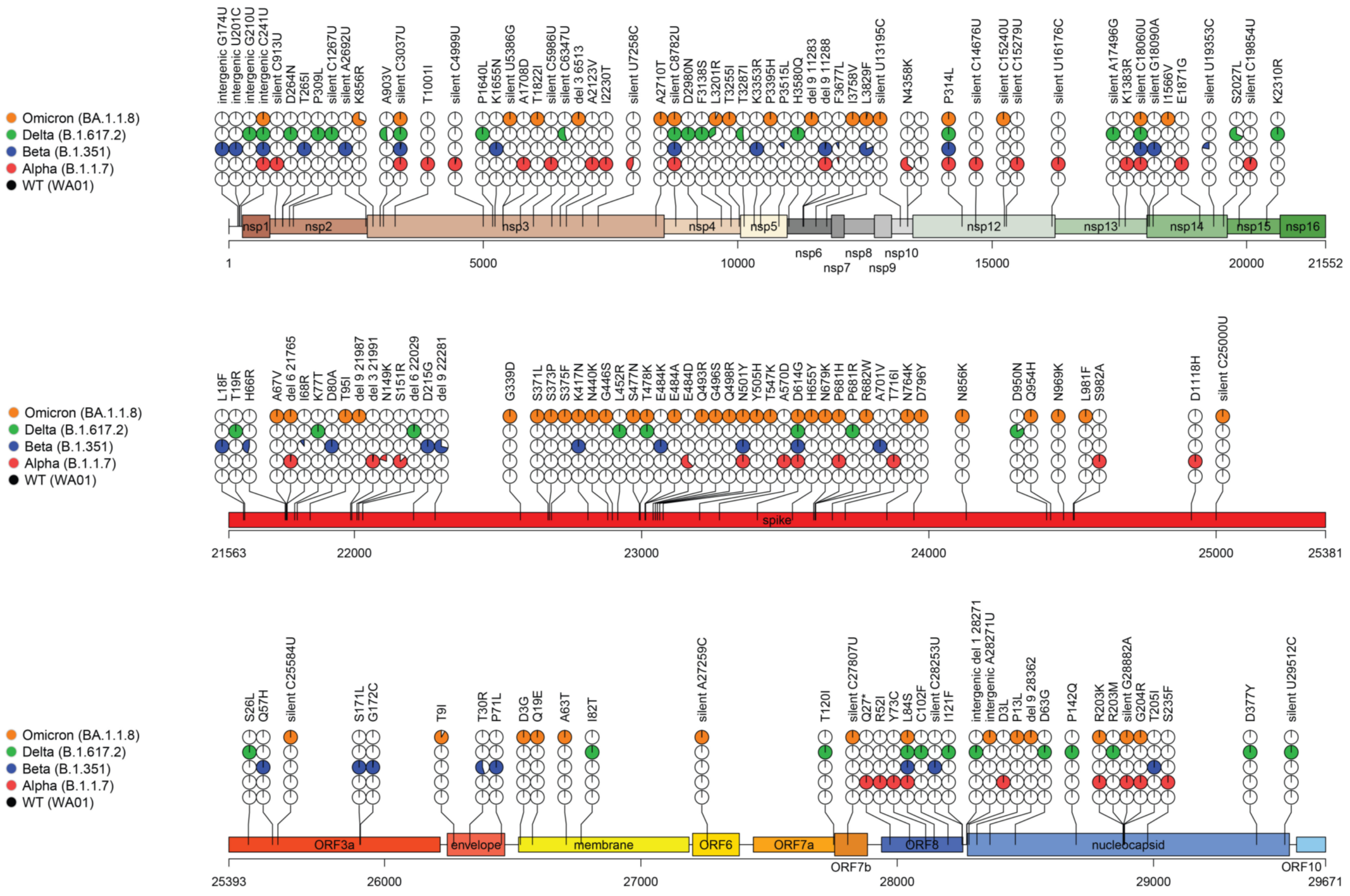

B

Counts of Samples by Variant and qPCR Protocol

Protocol	Alpha	Delta	Omicron	Other_Variant	PreVOC
Cepheid GeneXpert	15	68	50	6	4
Cobas 8800	4	622	557	7	3
Cobas Liat	0	25	51	0	0
DiaSorin MDX	3	11	0	0	0
Saliva COVID	10	140	473	3	4
ThermoFisher Amplitude	13	317	324	10	2

Fig S2

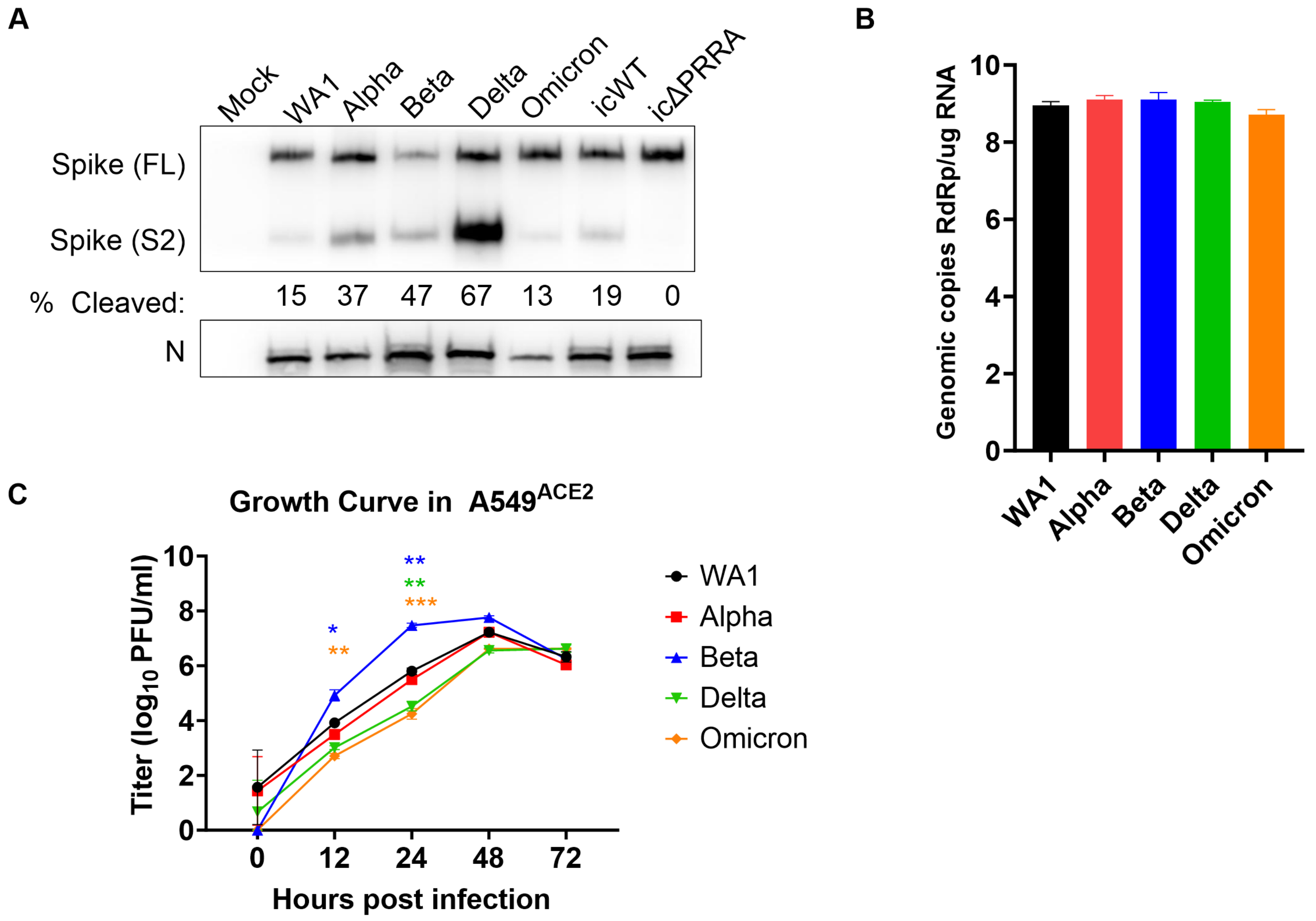
A
Mock
WA1
Alpha
Beta
Delta
Omicron
icWT
icΔPRRA
Spike (FL)
Spike (S2)
% Cleaved:
15
37
47
67
13
19
0
N
B
Genomic copies RdRp/ug RNA
10
8
6
4
2
0
WA1
Alpha
Beta
Delta
Omicron
C
Growth Curve in A549ACE2
Titer (log10 PFU/ml)
10
8
6
4
2
0
*
**
**
**
***
0
12
24
48
72
Hours post infection
WA1
Alpha
Beta
Delta
Omicron

Fig S3

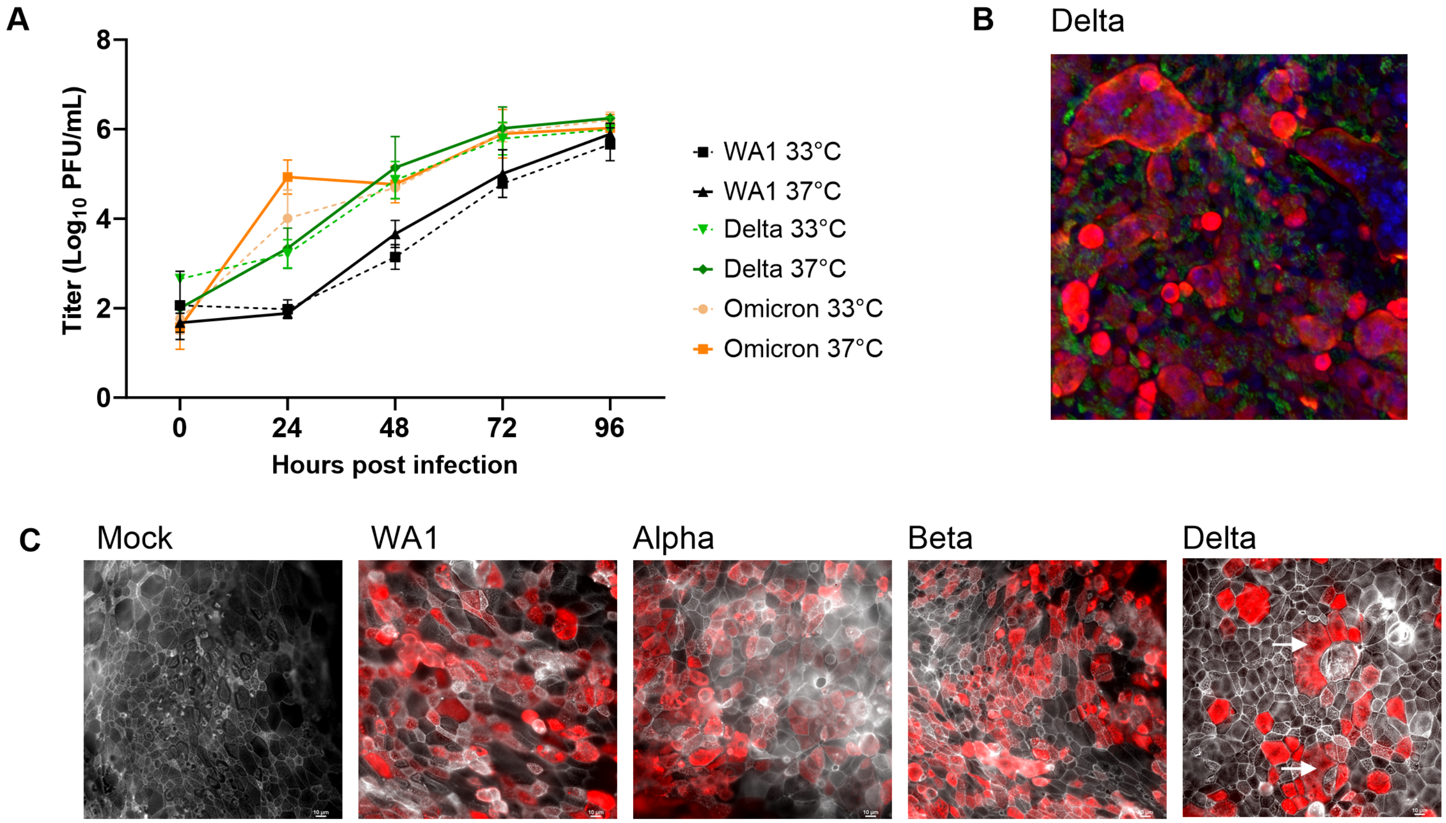
A
B
Delta
8
6
4
2
0
Titer (Log_{10} PFU/mL)
0
24
48
72
96
Hours post infection
WA1 33°C
WA1 37°C
Delta 33°C
Delta 37°C
Omicron 33°C
Omicron 37°C
C
Mock
WA1
Alpha
Beta
Delta

Table S1: Significant ISGs

WA1	Alpha	Beta	Delta	Omicron
CMPK2	BST2	CMPK2	BST2	BATF2
CXCL10	CMPK2	CXCL10	CMPK2	BST2
EPSTI1	CSF1	DDX60	CXCL10	CMPK2
HELZ2	CXCL10	EPSTI1	CXCL11	CXCL10
IFI44	CXCL11	IFI27	EPSTI1	CXCL11
IFI44L	DDX60	IFI35	HERC6	DDX60
IFIT1	EPSTI1	IFI44	IFI27	DHX58
IFIT2	HERC6	IFI44L	IFI35	EIF2AK2
IFIT3	IFI27	IFIT1	IFI44	EPSTI1
ISG15	IFI35	IFIT3	IFI44L	GBP2
LAMP3	IFI44	IFITM1	IFIT1	GBP4
OASL	IFI44L	IRF7	IFIT2	HELZ2
RSAD2	IFIH1	ISG15	IFIT3	HERC6
SAMD9L	IFIT1	LAMP3	IFITM1	IFI27
	IFIT2	OAS1	IRF7	IFI35
	IFIT3	OAS3	ISG15	IFI44
	IFITM1	OASL	LAMP3	IFI44L
	IFITM3	RSAD2	MX1	IFIH1
	IRF7		OAS1	IFIT1
	ISG15		OAS2	IFIT2
	LAMP3		OAS3	IFIT3
	MX1		OASL	IFITM1
	OAS1		RSAD2	IFITM3
	OAS2			IRF7
	OAS3			IRF9
	OASL			ISG15
	RSAD2			ISG20
	SAMD9L			LAMP3
				LY6E
				MX1
				NCOA7
				NMI
				OAS1
				OAS2
				OAS3
				OASL
				PARP12
				PARP9
				PLSCR1
				RSAD2
				RTP4
				SAMD9
				SAMD9L
				SLC25A28
				SOCS1
				SP110
				STAT1
				TAP1
				TRIM21
				UBE2L6
				USP18
